# The CARESSES study protocol: testing and evaluating culturally competent socially assistive robots among older adults residing in long term care homes through a controlled experimental trial

**DOI:** 10.1186/s13690-020-00409-y

**Published:** 2020-03-20

**Authors:** Chris Papadopoulos, Tetiana Hill, Linda Battistuzzi, Nina Castro, Abiha Nigath, Gurch Randhawa, Len Merton, Sanjeev Kanoria, Hiroko Kamide, Nak-Young Chong, David Hewson, Rosemary Davidson, Antonio Sgorbissa

**Affiliations:** 1 University of Bedfordshire, Park Square Campus, Luton, LU1 3JU UK; 2 Department of Informatics, Bioengineering, Robotics and Systems Engineering, University of Genova, Via all’Opera Pia 13, 16145 Genova, Italy; 3 Advinia Health Care Limited LTD, 314 Regents Park Road, London, N3 2JX UK; 4 Nagoya University, Furocho, Chikusaku, Nagoya, Aichi 464-8601 Japan; 5 Japan Advanced Institute of Science and Technology, 1-1 Asahidai, Nomi, Ishikawa 923-1292 Japan

**Keywords:** Study protocol, CARESSES, Cultural competence, Social robotics, Culturally competent robots, Artificial intelligence

## Abstract

**Background:**

This article describes the design of an intervention study that focuses on whether and to what degree culturally competent social robots can improve health and well-being related outcomes among older adults residing long-term care homes. The trial forms the final stage of the international, multidisciplinary CARESSES project aimed at designing, developing and evaluating culturally competent robots that can assist older people according to the culture of the individual they are supporting. The importance of cultural competence has been demonstrated in previous nursing literature to be key towards improving health outcomes among patients.

**Method:**

This study employed a mixed-method, single-blind, parallel-group controlled before-and-after experimental trial design that took place in England and Japan. It aimed to recruit 45 residents of long-term care homes aged ≥65 years, possess sufficient cognitive and physical health and who self-identify with the English, Indian or Japanese culture (*n* = 15 each). Participants were allocated to either the experimental group, control group 1 or control group 2 (all n = 15). Those allocated to the experimental group or control group 1 received a Pepper robot programmed with the CARESSES culturally competent artificial intelligence (experimental group) or a limited version of this software (control group 1) for 18 h across 2 weeks. Participants in control group 2 did not receive a robot and continued to receive care as usual. Participants could also nominate their informal carer(s) to participate. Quantitative data collection occurred at baseline, after 1 week of use, and after 2 weeks of use with the latter time-point also including qualitative semi-structured interviews that explored their experience and perceptions further. Quantitative outcomes of interest included perceptions of robotic cultural competence, health-related quality of life, loneliness, user satisfaction, attitudes towards robots and caregiver burden.

**Discussion:**

This trial adds to the current preliminary and limited pool of evidence regarding the benefits of socially assistive robots for older adults which to date indicates considerable potential for improving outcomes. It is the first to assess whether and to what extent cultural competence carries importance in generating improvements to well-being.

**Trial registration:**

Name of the registry: ClinicalTrials.gov

Trial registration number: NCT03756194.

Date of registration: 28 November 2018. URL of trial registry record.

## Contributions to the literature

- The CARESSES trial is one of the largest studies into the effectiveness of socially assistive robots (SARs) in improving health-related outcomes for older adults to date. The trial builds on the existing limited but promising evidence-base.
- The trial explores the impact of cultural competence by exploring if and how SARs employing the CARESSES culturally competent artificial intelligence produce improvements in outcomes.
- The complex and ethically sensitive methodology will aid future studies aiming to evaluate the impact of SARs on health-related outcomes.
- The results will aid the development of policies on the use of artificial intelligence and robotics in such settings.

## Background

CARESSES (Culture-Aware Robots and Environmental Sensor Systems for Elderly Support) is a multidisciplinary, international project whose goal is to design, test and evaluate socially assistive robots (SARs) configured to assist older adults residing in long-term care homes in a culturally competent manner [1].

Culturally competent robots autonomously re-configure their way of interacting with a user in a way that is appropriate to the culture, customs, and preferences of the person they are assisting. They begin by understanding which culture, at a group-level, the user primarily identifies with and as such accesses a relevant cultural knowledge database. The robot uses this database (a hierarchically structured ontology employing causal Bayesian networks that express correlations between different concepts) as a basis of its verbal and non-verbal interactions but then adapts its understanding of the user’s individual preferences and values as it receives feedback from the user during an interaction [2]. For example, the robot may initially guess that an American individual values baseball and not rugby and thus offers to talk about baseball. However, if the user expresses their dislike for baseball and a preference for rugby, then the robot’s understanding will change accordingly and, during subsequent interactions, will be more likely to initiate conversations about rugby than previously. By leveraging the principles of cultural competence during interaction with users, it is hypothesised that users may be more likely to accept and value interactions with SARs compared to SARs that do not perform this. This is important given how critical user acceptance is for the successful implementation of any public health intervention [3].

Cultural competence is also a concept that is linked to improved health outcomes among patients particularly within the nursing literature [4–7]. Papadopoulos [4] defines cultural competency as the ability to respond effectively to people from different cultures and backgrounds that can subsequently assist healthcare professionals in the delivery of services that meet the cultural and communication needs of patients. This concept has never previously been implemented and evaluated within robotics despite its importance for enhancing patient-centred care.

The evidence to date regarding the effectiveness of SARs in improving health-related outcomes for older adults is promising. For example, Abdi et al’s [8] scoping review of 33 studies consisting of 1574 participants and 11 robots showed that 28 of the 33 papers reported positive outcomes (five studies showed non-significant findings), and that SARs could be employed to offer a wide range of roles within the elderly care context particularly cognitive training, social facilitation, and companionship, for which findings were consistently positive. The authors also found SARs could also be useful in providing physiological therapy and affective therapy. However, for the latter, the findings were not clearly better than a comparative soft-toy or placebo robot, while for physiological therapy the results were sometimes clinically unpredictable. They conclude that the potential for SARs for improving outcomes is promising but that further evidence is required. A systematic review and meta-analysis of the effectiveness of SARs for older adults by Pu et al. [9] made similar conclusions. Their review synthesised evidence from 11 randomised controlled trials of SAR interventions for older adults, observing that SARs produced positive impact on agitation, anxiety, and quality of life for older adults, as well as indications that social robot interactions could improve engagement, interaction, and reduce loneliness. Due to such observations as well as concerns over study quality, the authors conclude that “the potential for social robots to improve cognition, depression, and apathy needs further investigation” (p13).

The CARESSES study is formed on the points and issues highlighted above, namely that it is reasonable to expect that leveraging cultural competence within socially assistive robots may yield particularly positive improvements in user outcomes and acceptance, and that there is a clear need for further research of SARs within the context of supporting older adult care. The need for effective, safe and acceptable interventions is underlined by the aging populations across the world. For example, it is estimated that by 2030, approximately one in five people in developed countries will be aged over 65 years, and that the population of over 85 years olds will almost triple by 2050 [10]. Such demographic changes are putting increasing pressure on health and social care systems especially regarding employee turnover and shortages [11]. In the UK alone, it is anticipated that over two million new workers will need to be trained and recruited into the health and social care sector between 2012 and 2022 [12].

The CARESSES project began in January 2017 and consists of a range of different work packages led by different research partners across Europe and Japan. The current protocol only describes the procedures used for testing work package (that was completed in November 2019) and also the evaluation work package (due to be completed in early 2020). The overall aim of these work packages are to conduct and evaluate a controlled before and after experimental trial aimed at exploring if and to what extent SARs employing the CARESSES culturally competent artificial intelligence can produce better health and well-being related outcomes among older adults residing in long stay care homes (as well as their informal carers) compared to a control robot, and care as usual (no robot intervention). The control robot possesses all of the same functionalities of the CARESSES robot but employs cultural competence in what the study team believe to be a less valid and reliable way. The specific objectives are:

1. Whether and to what degree improvement in health and well-being related outcomes are observed among participants in the experimental group, compared to those in the control groups.
2. Whether and to what degree the CARESSES robot was assessed by participants as more culturally competent than the CARESSES control robot.
3. To investigate participants’ views of the CARESSES robot’s cultural competence, including how and why they think the robot impacted their well-being, and whether and why they accepted the robot and its assistance.
4. To assess cost-effectiveness of the intervention compared to costs of care alternatives

## Methods

### Study design

A mixed-method, single-blind, parallel-group controlled before-and-after experimental trial with 1:1 participant allocation was employed between March and November 2019 within eligible long-term older adult care homes in England and Japan. The care homes were predominantly those owned by Advinia Health Care (a full research partner in the project) from which English and Indian residents were recruited, and also within the HISUISUI assisted living facility in Japan from which Japanese residents were recruited. The testing procedures took place within participants’ bedrooms only. A pre-trial pilot study was successfully employed in November and December 2018 in one UK-based care home that assessed the feasibility, acceptability and fidelity of the intervention and its procedures. This led to a range of procedural and technical improvements made prior to the commencement of the full trial.

### Participants

A total number of 45 residents of older adult long-term care homes, who self-identify themselves as primarily belonging to English (*N* = 15), Indian (N = 15) or Japanese (N = 15) cultures were aimed to be recruited within the UK (*N* = 30) and Japan (N = 15). The other eligibility criteria for participating residents were:

- Aged ≥65 years.
- Resided in a single occupancy bedroom / bedroom area.
- Unlikely to express aggression towards themselves, the robot, and/or the researcher.
- Possessed sufficient cognitive competence.
- Possessed sufficient physical health.
- Able to verbally communicate in English (UK site only) or Japanese (Japan site only).

To determine residents’ eligibility, a two-staged screening procedure was employed. During the first stage, care home staff nominated residents (using initials only to protect anonymity) who they believed met the inclusion criteria. Only staff who directly provided care to the residents and therefore possessed knowledge of each residents’ physical and cognitive competency were requested to make these nominations. During the second stage, the research team supported the nominating care home staff in using the following screening tools which establish participant eligibility. For assessing cognitive competence and aggressiveness, the interRAI-Long Term Care Facility ‘Cognitive Performance Scale’ and Aggressive Behaviour Scale’ sub-scales were used [13]. For the cognitive scale, scores range from 0 (intact) to 6 (very severe impairment), with a score of 3 or more indicating moderate to severe impairment. Therefore, given the need for reasonable cognitive understanding of how to use and interact with the robot, a threshold of ≤2 was set. For the aggression scale, a total score above 0 indicates a reasonable likelihood of aggression and therefore, to reduce this risk, a threshold of < 1 was set. For assessing frailty (a proxy to physical health), the FRAIL-NH scale [14] was employed with an applied threshold of ≤10. This has previously been recommended as a cut-off for grouping higher/lower frailty in the older adult population residing in long-term care home settings [15]. Residents who passed the second screening stage were then approached, with a researcher being introduced to the resident by a care home staff member at a convenient and appropriate time. Residents were not asked to consent to being nominated or screened during these stages because there was a reasonable likelihood that doing so could have caused confusion and upset to some residents. For example, the researcher may have caused a resident confusion and potentially distress had the resident misunderstood our request because of poor cognitive competence. Further, if a resident provided consent and was keen to participate but was subsequently not invited, it may have caused disappointment.

Approached residents were then provided a participant information document and informed consent document (produced following Care Quality Commission and Alzheimer Europe Ethical Guidelines recommendations) as well as a study leaflet that visually informed residents of the hardware and outlines some of its functionalities. The researcher then offered to read these documents aloud to the resident and encouraged him/her to speak with informal carers if they were interested in participating. If a resident wished to provide consent but could only do so through verbal means, then this was conducted in the presence of a literate witness who could countersign the consent document on behalf of the participant. A rolling consent approach was also adopted throughout the study to help ensure that participants may withdraw at any time. To incentivise participation, residents were offered a £30/¥4000 voucher if they were allocated to the care as usual group. Participants who received a robot were also provided with a printed photo album and videos of their time with the robot after their final testing period had been completed.

Residents who provided consent were asked to nominate up to three informal carers to participate and, if multiple informal carers were nominated, to indicate who they considered to be their primary informal carer. A researcher then contacted the primary informal carer (if applicable) to invite them to also participate. If he/she declined, another nominated informal carer was contacted and invited to participate. If three informal carers were nominated, the process would be repeated a final time if the second informal carer also declined. When a meeting to seek their consent to participate was arranged (which took place at a mutually convenient time either in-person or via a phone call or videoconferencing), a researcher provided the informal carer with a study leaflet, a participant information document and informed consent form (all tailored for their use). To incentivise participation, informal carers were offered a £30/¥4000 voucher.

Beyond being nominated, informal carers were eligible to participate if they met the following additional eligibility criteria:

- Aged ≥18 years.
- Had visited the participant in the care home within the past 3 months.
- Provided any type of informal help, care and/or support to the participant.
- Were a relative, partner, friend or neighbour who has a significant personal relationship with the participant.
- Were not paid or officially employed to provide care to the participant.
- Able to communicate in English (UK site only) or Japanese (Japan site only).

**Fig. 1:**
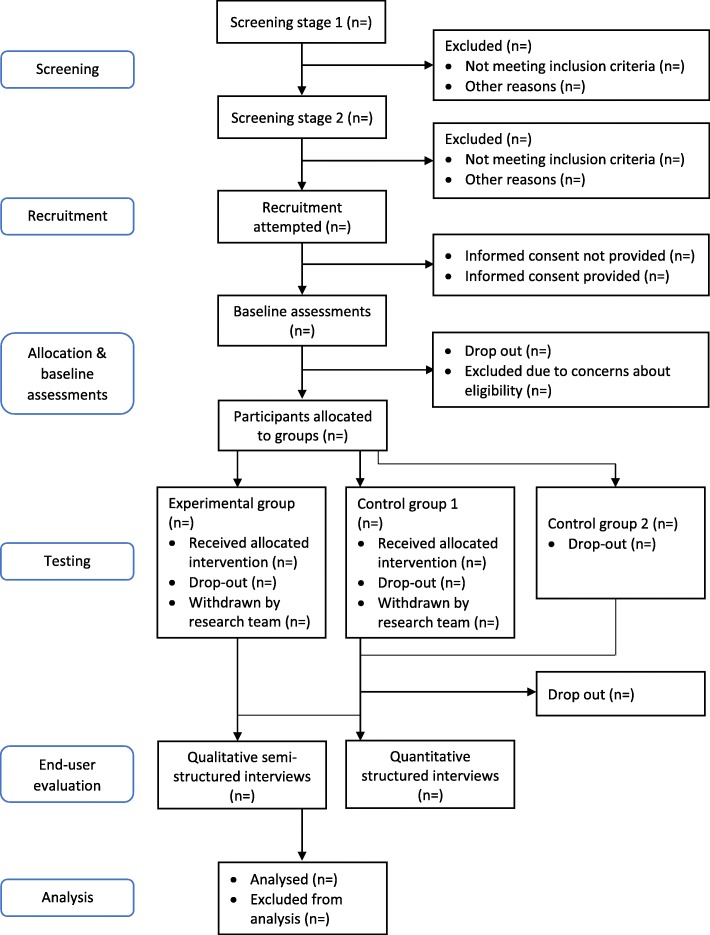
Study design flow chart

Due to financial, time and resource restrictions (UK: 20 weeks for testing procedures, two robots only; Japan: 12 weeks for testing procedures, three robots only), and the inability to statistically power this intervention (due to the novelty of the intervention and thus uncertainty over what might constitute a clinically meaningful difference in outcomes), it was unlikely to be possible to collect data from more than 15 participating care home residents per cultural group. See Fig. 1 and Table 1 for a breakdown of the recruitment procedure and study design.

**Table 1:** Study design overview

	English group	Indian group	Japanese group
**N participants (residents) total**	15	15	15
**N participants (informal carers) total**	15	15	15
**Testing site**	England	England	Japan
**Participant and intervention language used**	English	English	Japanese
**N participants (residents + informal carers) in experimental group**	5 + 5	5 + 5	5 + 5
**N participants (residents + informal carers) in control group 1**	5 + 5	5 + 5	5 + 5
**N participants (residents + informal carers) in control group 2**	5 + 5	5 + 5	5 + 5
**Duration of robot interaction testing period (for residents in experimental or control group 1)**	2 weeks: • Week 1: 3 days, 45 min - 3 h per day (rotated). First session is training, all other sessions independent use with standby support ready. • Week 2: 3 days, 45 min - 3 h per day (rotated) – Independent use with standby support ready.		
**Total time required for testing**	20 weeks		12 weeks

### Allocation and blinding

Participants were allocated to one of three study groups using random sampling stratified by gender. The study groups were as follows: experimental group (utilizing a CARESSES experimental robot), control group 1 (utilizing a CARESSES control robot) and control group 2 (care as usual without robot intervention). Participants (both residents and their informal carers) who received a robot were blinded to which type of robot they received (the experimental or control robot). Care home staff were also blinded and there were no circumstances under which unblinding was permissible.

### Trial preparation

#### Training and supporting research staff

Prior to trial commencement, all research staff were subject to Disclosure & Barring Service checks (in the UK) and Criminal Record checks (in Japan). Research staff also completed ethics training (from the project’s internal ethics board), methods training (from CP) and pre-selected relevant online courses produced by Advinia Health Care (in the UK site) and by HISUISUI (in Japan site) for their care home staff. Staff also had weekly supervisory meetings, encouraged to keep a personal reflective diary, and reminded to contact project colleagues for additional support during the implementation of procedures in relation to ethical, methodological and/or technical issues and queries that emerged.

#### Care home staff preparation

A brief presentation was arranged with care home staff at a convenient and appropriate time to prepare them for what to expect during the testing procedures and to request that they continue their jobs as usual and to not rely upon the robot for any reason or change the usual provision and standard of care provided. This was to reduce the risk of confounding outcomes (e.g. because of a potential drop or change in the way that participants received care from their staff) and for ethical reasons (e.g. a reliance on the robot or decline in provision/quality of care could negatively affect participants). To help protect staff morale and concerns about the implications of technological interventions, staff were reminded that CARESSES is about understanding whether and how such interventions may improve outcomes in conjunction with current care rather than replacing care. Leaflets that conveyed this key information were distributed during the presentation and left in the main staff office for staff members who are unable to attend.

#### Pre-trial meeting with participants

A brief informal meeting was arranged with participants allocated to the experimental group or control group 1 to collect key personal information required to customise the robot including name, age and the names and contact information of informal carers that the robot could use to contact if called upon by the resident. A reminder card was also provided that stated when the first testing session shall take place. Any remaining questions prior to the first testing session were also addressed.

#### Technical preparation

Technical preparation and set-up procedures took place within each participant’s bedroom. This was necessary to install the required equipment (including networking, the robot, a small docking station, a video camera and microphone for monitoring), to map the bedroom space and to make note of objects of relevance (e.g. wardrobe) that the robot may draw attention to during interactions as appropriate. This required approximately 1 h to complete and was conducted in the presence of the resident or without their presence if they prefer and provide consent for. The specific robot hardware used in the study was the Pepper robot; a human-shaped robot manufactured by SoftBank Robotics. It is 4 ft tall and weighs 63 pounds.

#### Boosting standardisation between sites

To boost standardisation of procedures between the UK and Japan based sites, a number of procedures took place. First, a highly detailed protocol had been produced between the lead partners of both sites. In addition, to aid the development of the protocol, informal visits of candidate care homes in different sites had been made by the lead partners of both sites. Third, pre-existing validated Japanese translations of research instruments had been selected where possible, with all other relevant documents undergoing professional translation. Fourth, researchers from both sites had undergone the same ethics and methods training procedures and took part in a number of additional preparatory meetings, both in person (during yearly pre-planned project meetings) and online using Zoom videoconferencing software. Fifth, the Japanese procedures commenced 3 months after the commencement of procedures in the UK. This helped to ensure that any unanticipated required procedural changes could be considered and planned for prior to Japanese testing. Finally, step-by-step manuals to aid implementation of technical and methodological procedures between sites had also been produced.

### Interventions

#### Experimental group – CARESSES experimental robot

Participants allocated to this group received a CARESSES experimental robot for 2 weeks. This robot was culturally aware and thus aware of the resident’s cultural background prior to testing commencing. Therefore, it pre-loaded the appropriate Cultural Knowledge Base (CKB) and applied it during its time with the resident. During the first week, the robot interacted and provided culturally competent assistance on a culture-specific level only i.e. it did not learn and adjust to the participants’ individual values and preferences. In week 2, the robot was configured to be able to learn and adapt to the individual’s cultural profile thus moving from a culture-specific approach to a personalised approach. Its learning included ‘propagation’ i.e. personalised adjustments to the CKB in one area automatically lead to appropriate knowledge adjustments in other related areas. The specific functionalities of the robot included ‘chit-chatting’ (conversations about topics of interest and cultural relevance), setting reminders, playing music and videos, reading out audiobooks, making audio or video calls to informal carers and sending text messages and pictures to them, and contacting formal care staff members. It could also offer privacy (by turning away from the resident and looking down at the floor), display a news or weather report, suggest dressing and clothing options (moving to the wardrobe, talking about clothes, showing pictures of clothes, showing videos of wearing clothes), assisting with religion and prayer (reminding them to take the required objects, suggesting an appropriate place, reminding them what rituals are), assisting with meals (talking about food, showing images of meals, showing care home menus), control a smart light, play simple games, tell jokes, and enter a relaxation mode (relaxing music and relaxing scenes).

#### Control group 1 – CARESSES control robot

Participants allocated to control group 1 received a CARESSES ‘control’ robot for 2 weeks. This robot was not aware of the resident’s cultural background prior to the testing commencing. Rather, it pre-loaded a generic and more limited CKB that was not tailored for any particular culture-specific profile. In week 1, the robot did not learn and adjust to the participants’ individual cultural values and preferences. In week 2, however, the robot was configured to be able to learn and adapt to the individual’s cultural profile thus moving to a personalised approach. This robot’s learning did not include propagation but did possess the same suite of functions as the CARESSES robot although was less likely, in theory, to offer these in a culturally competent way. Because of the novelty of this type of research experiment, it remained unknown as to whether limiting the CKB, preventing propagation of learning and being culturally non-aware at the outset of interactions would indeed lead to poorer outcomes compared to the CARESSES robot (thus ensuring clinical equipoise).

#### Control group 2 – care as usual

Participants allocated to this group did not receive a robot and continued to receive care as usual.

#### Testing procedures

Each participant allocated to the experimental group or control group 1 had six sessions with the robot, each of which may lasted for up to 3 h. Sessions 1–3 were spread across week 1 (e.g. Monday, Wednesday, Friday) and sessions 4–6 were spread across week 2. The specific timings of these sessions were discussed and agreed with by the resident so that they took place at convenient times that, as much as possible, did not conflict with pre-existing planned activities. If a session lasted for less than 45 min, then this was considered a part 1 of 2 session. However, because of logistical and timing constraints, no more than 2 parts of one session could take place even if part 2 again lasted for less than 45 min. A minimum session threshold of 45 min was set because this was considered to be, theoretically, a reasonable minimum amount of time for a participant to potentially benefit from having access to the robot (an estimation partly based on the pre-trial pilot).

Session 1 consisted of a training session during which a researcher informed and guided a participant on how to use the robot and its functionalities (including what the robot can and cannot do), how best to communicate with the robot and how and when to request assistance. The remaining 5 sessions allowed the participant to use the robot independently and privately although all sessions were audio-video monitored for safety and scientific purposes (which participants were aware of). For these sessions, a researcher may have been present if requested by the participant and until he/she felt comfortable with interacting independently. Participants were also reminded that they may use the robot as much or as little as they wish and that they were free to leave their bedroom area for as long as they wish. A session was ‘live’ so long as the system was not suspended or turned off (due to technical problems, a desire by the participant to switch the system off or because the participant starts an activity which the research team considers unethical to continue to visually monitor).

During all sessions, a researcher and technician was present outside the participants’ bedroom to provide guidance and support when requested, and to intervene if the monitoring process identified any technical problems or concerns that may have jeopardised the participant’s safety and well-being.

After the final session was completed, the participants were debriefed and thanked. If the participant expressed sadness, upset and/or distress, the researcher provided company and support until their well-being had been observed to improve. In addition, a researcher also telephoned each participant 1 and 3 weeks after testing was completed for an informal conversation in part to assess whether any sadness or distress as a result of missing the robot had remained, in which case this was brought to the attention of the appropriate formal carer. Researchers were provided support to conduct these procedures during their ethics and methods training sessions, and had access to additional ongoing support and guidance from their supervisors (CP, LB).

### Data collection

Research measurements were performed during baseline (T0), after 1 week of testing (T1) and after 2 weeks of testing (T2) among resident participants and informal carers. The specific instruments that were employed (via quantitative structured interviews in all cases) were:

- Cultural Competence Assessment Tool – Robotics (CCATool-Robotics). This is an adapted version of the RCTSH Cultural Competence Assessment Tool (CCATool) [16]. The tool measures older adults’ perceptions of the robot’s cultural awareness, cultural knowledge, cultural sensitivity, and cultural competence. This was completed by resident participants who receive a robot during T1 and T2.
- Short Form (36) Health Survey version 2 (SF-36 v2). The SF-36v2 [17] is a widely used, reliable and validated multi-purpose, short-form health survey with 36 questions. It measures the following eight dimensions of health: general health, bodily pain, emotional role limitation, physical role limitation, mental health, vitality, physical functioning and social functioning. This was completed by all resident participants and informal carers during T0 and T2.
- Short Form University of California Los Angeles (UCLA) Loneliness Scale (ULS-8). Loneliness was measured using the widely used and validated Short-Form Measure of Loneliness [18]. The scale is comprised of eight items to assess loneliness using a 4-point Likert scale with values ranging from “never” to “always”. This was completed by all resident participants and informal carers during T0 and T2.
- Negative Attitudes towards Robots Scale (NARS). This scale assesses participants’ attitudes towards robots [19]. NARS is comprised of 14 items scored on a 5-point agreement Likert Scale which measure three attitudinal domains: ‘Situations of interaction with robots’, ‘Social influence of robots’ and ‘Emotions in interaction with robots’. This was completed by all resident participants and their informal carers during T0 and T2.
- The Zarit Burden Inventory (ZBI). The ZBI consists of 22-items on a 5-point Likert Scale that measure subjective care burden among informal carers [20]. Its validity and reliability have been widely established. The scale items examine burden associated with functional / behavioural impairments and care situations. This was completed by all informal carers during T0 and T2.
- Questionnaire for User Interface Satisfaction (QUIS). The QUIS instrument uses a 9-point scale to measure users’ overall satisfaction of a technological system and its interface [21]. Some of the original statements were amended to assess the ease of use and usability of a robot. Thiswas completed by resident participants who received a robot during T2.

Qualitative one-to-one semi-structured interviews were also conducted with residents, and their informal carers, allocated to receive a robot. An interview schedule had been designed to elicit discussions related to: perceptions of the robot’s cultural competence, acceptability of and satisfaction with the robot’s interactions, quality of service provided, and impact the robot had upon their health and well-being, independence and autonomy. The interviews were conducted by a researcher who was already familiar with the participant. The researcher requested that the interview was audio-recorded (although this is not a requirement) and ensured that it took place at a convenient time for the resident or informal carer. For the latter, the option for interviews to take place over video-call or telephone were also made. Observations and reflections by the project team regarding the level of technical success, as well as any methodological/procedural or ethical issues and deviations during the study process were also collected and discussed during supervisory meetings or earlier if required with the project team.

### Data analysis

This work underpins the evaluation work package of the CARESSES study which shall be completed in early 2020. Quantitative data will be analysed using the IBM SPSS statistical software version 21.0 [22]. Kolmogorov-Smirnov tests will be initially conducted to establish the normality of the dependent variables. As appropriate, independent samples t-tests (for normal distribution), Mann-Whitney U tests (for non-normal distribution), or Chi-Square tests (normal or non-normal) will be used to compare the independent and dependent variables between the two groups/arms, in addition to simple descriptive statistics (means, medians and SDs as appropriate). For within-group repeated analyses, dependent t-tests (for normal distribution) and Wilcoxon signed-rank tests (for non-normal distribution) will be used to assess changes between pre-test and post-test scores. The level of statistical significance will be set at *p* ≤ 0.05. Linear mixed models will be used to assess the magnitude of differences in changes between the experimental and control groups. Drop out and intention-to-treat analyses will also be conducted. Fidelity will be assessed through a fidelity implementation checklist to be completed by the research team as the procedures take place. To assess cost-effectiveness, the EU MAFEIP (Monitoring and Assessment Framework for the EIP on Active and Healthy Ageing) tool will be used to estimate lifetime incremental quality-adjusted life years (using SF-36 data) with incremental cost of our intervention (compared against costs of care alternatives).

Qualitative data will be transcribed verbatim, entered into NVivo software Version 10.0 [23] and analysed using thematic analysis as described by Braun and Clarke [24]. This will involve two researchers independently reading and re-reading the transcripts to familiarize themselves with the data and independently labelling the data into categories and codes. Meaningful patterns across all the codes that are identified will be sorted into themes. All the codes under each theme will then be reviewed to confirm the validity of each theme and to produce overarching themes.

### Ethics and data management

The Ethical Guidelines of Alzheimer Europe for older adults with mild cognitive impairment have been used to shape the assessment and management of the key ethical issues inherent in this study. After conducting thematic analysis on this, nine broad ethical themes were identified as relevant and addressed, namely: attachment, authentic interaction and reciprocity; substitution for social contact; autonomy; culturally determined values and preferences; dignity and personhood; privacy; informed consent; preventing harm; and stigma. A full explanation of how these themes were identified within the trial is described elsewhere [25]. Protocols for the management of distress, incidental findings and reportable events have also been produced. Protocol modifications were discussed, recorded, justified and communicated with the Research Ethics Committees if deemed necessary.

The project committed to the maintenance of participants’ anonymity and confidentiality throughout all procedures, including screening, recruitment, testing, evaluation and dissemination procedures. Data collection, usage and storage procedures complied with national laws and the EU’s General Data Protection Regulation (GDPR) including the commitment of participants’ the right to access, right to be informed, right to withdraw and right to data erasure. Data collection complied with the principle of data minimization i.e. that the collection of personal information from study participants is limited to what is directly relevant and necessary to accomplish the specific goals of the testing and evaluation work packages. No data related to a third party was stored; this included any audio, video or sensory data collected upon a person, not part of the study, such as a visitor or a staff member, who entered a bedroom during testing. The editing process of the video clips provided to participants at the end of testing procedure included ensuring that non-participants had their identity protected through blurring faces. All screening data was discarded upon completion of the project. During the testing procedures, all visual, auditory and sensory data that the robot collects and processes in order to function as planned is discarded after the procedures have been completed. The exception to this was the collection of the number of interactions that the robot logs with each participant. However, these interactions were anonymous. Quantitative and qualitative research data were entered, stored and managed online through an encrypted and secure Google Drive project account with only project team members having access, and CP leading on data monitoring. All research data shall be made openly available for secondary analysis 3 years after the project has been completed.

## Discussion

The CARESSES research trial is one of the largest studies of its kind, particularly in terms of evaluating the potential benefits of socially assistive robots to the health and well-being of older adults residing in care settings, including their informal carers. It is also the first to assess whether and to what extent cultural competence carries importance in generating improvements to well-being. The trial will also add to the evidence base relating to the understanding of the user’s lived experiences of being supported by socially assistive robots, as well as user acceptance and attitudes towards artificial intelligence and robotic technologies. The results of this trial will also aid the development of policies for the use of artificial intelligence and robotics for older adults residing in long term care settings. However, it is important to re-emphasise that the CARESSES research trial is centred on understanding the impact this type of intervention may have in conjunction with existing care rather than in place of such care.

With social care struggling to cope with the growing demands of ageing populations across the world, and preliminary evidence of socially assistive robotic technologies indicating positive outcomes, implementing the CARESSES project in as rigorous and ethical a way as possible carries moral importance. However, it is important to emphasise that as this trial lacks statistical power and is based on only *n* = 45 participants across two sites, the results of this should be viewed as preliminary and indicatory only. Further rigorous research into the impact of such technologies, including their clinical efficacy, cost-effectiveness, sustainability, ethical implementation including impact upon staff morale (which is of critical importance and must be prioritised), will continue to be needed regardless of this trial’s findings.

## Data Availability

Not applicable.

## Abbreviations

ABS: Aggressive Behaviour Scale
CARESSES: Culturally-Aware Robots and Environmental Sensor Systems for Elderly Support
CCATool-Robotics: Cultural Competence Assessment Tool – Robotics
CKB: Cultural Knowledge Base
FRAIL-NH: Fatigue, Resistance, Ambulation, Incontinence, Loss of weight, Nutrition approach and Help with dressing screening tool
TPS: Technical Problems Scale
GDPR: General Data Protection Regulation
NARS: Negative Attitudes towards Robots Scale
QUIS: Questionnaire for User Interface Satisfaction
SARs: Socially-Assistive Robots
SF-36: Short Form (36) Health Survey
UCLA ULS-8: Short form University of California Los Angeles Loneliness Scale
ZBI: The Zarit Burden Inventory
